# Regional white matter hyperintensity volume predicts persistent cognitive impairment in acute lacunar infarct patients

**DOI:** 10.3389/fneur.2023.1265743

**Published:** 2023-10-10

**Authors:** Tan Li, Mengfan Ye, Guopeng Yang, Shanshan Diao, Yun Zhou, Yiren Qin, Dongxue Ding, Mo Zhu, Qi Fang

**Affiliations:** ^1^Department of Neurology, The First Affiliated Hospital of Soochow University, Suzhou, Jiangsu, China; ^2^Suzhou Jiasheng Medical Instrument Co., Ltd., Suzhou, Jiangsu, China; ^3^Department of Radiology, The First Affiliated Hospital of Soochow University, Suzhou, Jiangsu, China

**Keywords:** regional white matter hyperintensity volume, persistent cognitive impairment, acute lacunar infarct, cerebral small vessel disease, neuropsychology

## Abstract

**Background:**

White matter hyperintensity (WMH) is often described in acute lacunar stroke (ALS) patients. However, the specific relationship between regional WMH volume and persistent cognitive impairment remains unclear.

**Methods:**

We enrolled patients with ALS who were hospitalized at the First Affiliated Hospital of Soochow University between January 2020 and November 2022. All patients were assessed for global cognitive function using the Montreal Cognitive Assessment (MoCA) scale at 14 ± 2 days and 6 months after the onset of ALS. Manifestations of chronic cerebral small vessel disease (CSVD) were assessed via MRI scan. The distributions of regional WMH were segmented, and their relationship with cognitive impairment was evaluated.

**Results:**

A total of 129 patients were enrolled. Baseline frontal WMH volume (OR = 1.18, *P* = 0.04) was an independent risk factor for long-term cognitive impairment after ALS. Furthermore, the presence of WMH at the genu of the corpus callosum (GCC) at baseline (OR = 3.1, *P* = 0.033) was strongly associated with persistent cognitive decline. Multivariable logistic regression analysis showed that depression (OR = 6.252, *P* = 0.029), NIHSS score (OR = 1.24, *P* = 0.011), and albumin at admission (OR = 0.841, *P* = 0.032) were also important determinants of long-term cognitive impairment after ALS.

**Conclusions:**

Our study found that WMH, especially frontal WMH volume and the presence of WMH at the GCC at baseline, independently contributed to long-term cognitive decline in ALS patients. This study provides new evidence of the clinical relationship between regional WMH volume and cognitive impairment in ALS patients.

## Introduction

Acute lacunar stroke (ALS) accounts for nearly 25% of all ischemic strokes (1). Cognitive impairment is common after stroke. The volume and location of the lesions, related vascular risk factors, and probable underlying mechanisms all vary among the different stroke subtypes, which may influence the occurrence and severity of post-stroke cognitive impairment (PSCI). Stroke attributed to large-vessel atherosclerosis is generally considered to carry a higher risk of cognitive impairment. However, lacunar stroke with small-artery occlusion may pose a higher risk of PSCI than expected, with affected patients having an 11%−23% risk of developing dementia (2, 3), which is close to the risk level in other stroke subtypes (4). Additionally, cognitive impairments in lacunar stroke patients affect global cognition rather than specific domains (5). Lacunar stroke patients with cognitive impairment are also at a higher risk of unfavorable prognosis, recurrent stroke, and death (6).

Lacunar stroke is one of the subtypes of cerebral small vessel disease (CSVD), which is a systemic pathology of the whole brain (7). Manifestations of CSVD, such as lacunar stroke and white matter hyperintensity (WMH), have been recognized as leading risk factors for cognitive impairment (8, 9) and dementia (10, 11) in older people. Furthermore, CSVD is also an important risk factor for early recurrence of stroke after a TIA or stroke (12). Patients with ALS usually have a relatively high CSVD burden (13) compared to other elderly populations. The presence of cognitive impairment in lacunar stroke patients probably indicates advanced microangiopathy, which might be worsened by preexisting forms of CSVD, such as WMH, enlarged perivascular space (EPVS), cerebral microbleed (CMB), or brain atrophy (14).

In previous studies, many researchers have investigated the effects of total WMH burden or total WMH volume on cognition (15–18). Recently, the effect of WMH on cognitive function has been hypothesized to differ according to the regional distribution of WMH. Biesbroek et al. (19) have also suggested that the location of WMH is a determinant of the nature of cognitive impairment. The regional specificity of WMH has rarely been investigated. S. Kim et al. (20) found that periventricular WMH adjacent to the lateral ventricles might play a larger role in all-cause dementia (ACD) than deep WMH. Regarding brain lobes, one study has reported that WMH in the frontal, occipital, and temporal regions is associated with cognitive dysfunction in patients suffering from Alzheimer's disease (21). Rizvi et al. (22) also found that higher WMH volumes in the frontal, parietal, and occipital lobes were associated with poorer memory performance in a sample of healthy older adults. However, few studies have focused on the relationship between the regional lobar distribution of WMH and cognitive impairment in ALS patients. In this study, we aimed to assess the influence of the volume distribution of WMH on long-term cognition following ALS.

## Materials and methods

### Study design and participants

This prospective study was conducted at the Department of Neurology at the First Affiliated Hospital of Soochow University. We consecutively recruited patients who were admitted to our department between January 2020 and November 2022 with an imaging-proven lacunar infarction. Acute lacunar stroke was defined as an acute ischemic lesion with a diameter of ≤ 20mm on diffusion-weighted imaging (DWI) sequence in the perforator artery supply area, with its TOAST classification considered to be small artery occlusion, which has also reported in previous articles (23, 24). We excluded patients to whom any of the following criteria applied: (1) a cortical and/or cortico-subcortical non-lacunar infarct (an axial diameter of > 20 mm on diffusion-weighted MRI sequences); (2) macroangiopathy or cardioembolism; (3) preexisting disability [modified Rankin Scale score (mRS) >1]; (4) a history of severe depression (17-item Hamilton Depression Rating Scale score≥24) or other mental illness; (5) cognitive disorder attributed to severe systemic or other diseases; (6) WMH due to other specific causes; (7) conditions causing inability to cooperate with the clinical assessment, such as disturbance of consciousness, severe language disorders, or blindness; or (8) inability to undergo complete MRI scanning. The study was approved by the ethics committee of the First Affiliated Hospital of Soochow University (approval number 222315), and all participants gave written informed consent.

### Data acquisition

Demographic and clinical baseline data were collected within 48 h of admission; these included age, gender, education level, previous medical history [stroke, hypertension, diabetes mellitus, dyslipidemia, and coronary heart disease (CHD)], baseline National Institutes of Health Stroke Scale (NIHSS) score, baseline mRS score, baseline blood glucose and lipid levels, homocysteinemia, albumin, fibrinogen, and other relevant blood indexes.

### Neuropsychological assessment and follow-up visit

Neuropsychological tests were conducted by two experienced investigators (SSD and YZ) certified to administer neuropsychological assessments at 14 ± 2 days after stroke onset. The Montreal Cognitive Assessment (MoCA) has high sensitivity and specificity for mild cognitive impairment. According to the guidelines from the Vascular Impairment of Cognition Classification Consensus Study (VICCCS) of the Chinese Revision Plan, cognitive impairment is defined as follows: for individuals with no education, a MoCA score of ≤13 is indicative; for those with 1–6 years of education, a MoCA score of ≤19 is indicative; and for those with 7 or more years of education, a score of ≤24 is indicative (25). Additionally, the 17-item Hamilton Depression Scale (HAMD-17) was used to assess depression severity. A score of < 7 indicates a normal condition (26). A follow-up visit, including neuropsychological evaluations, mRS score assessment, and medication status, was conducted at 6 months (day 182 ± 7) after stroke onset in the form of an outpatient visit or by video. None of the physicians knew the baseline information about the patients.

### Brain MRI

All patients underwent a brain MRI scan (Achieva, PHILIPS Medical Systems). We acquired WMH volume using axial 2D T2-fluid-attenuated inversion recovery (T2-FLAIR) images (8000/120 ms for TR/TE; FOV: 220 × 175 × 119 mm; section thickness: 5 mm). Other sequences employed in the study included DWI, 3d-TOF-MRA, T2-weighted, T1-weighted, and susceptibility-weighted imaging (SWI). A total CSVD burden score was calculated according to previously published methods; this consisted of four imaging markers, namely, silent lacuna, WMH, EPVS, and CMB (27). Briefly, WMHs were rated in the FLAIR sequence according to the Fazekas scale. One point was added to the total burden score for either severe periventricular WMH (Fazekas score 3, irregular extension into the deep white matter) or moderate-to-severe deep WMH (Fazekas score 2 or 3, early confluence or confluence). EPVS in the basal ganglia was rated on a semiquantitative scale (grade 0, no EPVS; grade 1, <10 EPVSs; grade 2, 11–20 EPVSs; grade 3, 21–40 EPVSs; grade 4, more than 40 EPVS), and one point was added for EPVS at level 2–4. We used the SWI sequence to evaluate the presence of deep CMBs in the basal ganglia, thalamus, brain stem, and cerebellum. One point was added for the presence of any lacuna or CMB. Total CSVD burden was graded into three levels of severity: mild (0 or 1 point), moderate (2 points), or severe (3 or 4 points) (28, 29).

### WMH segment

**Figure 1:**
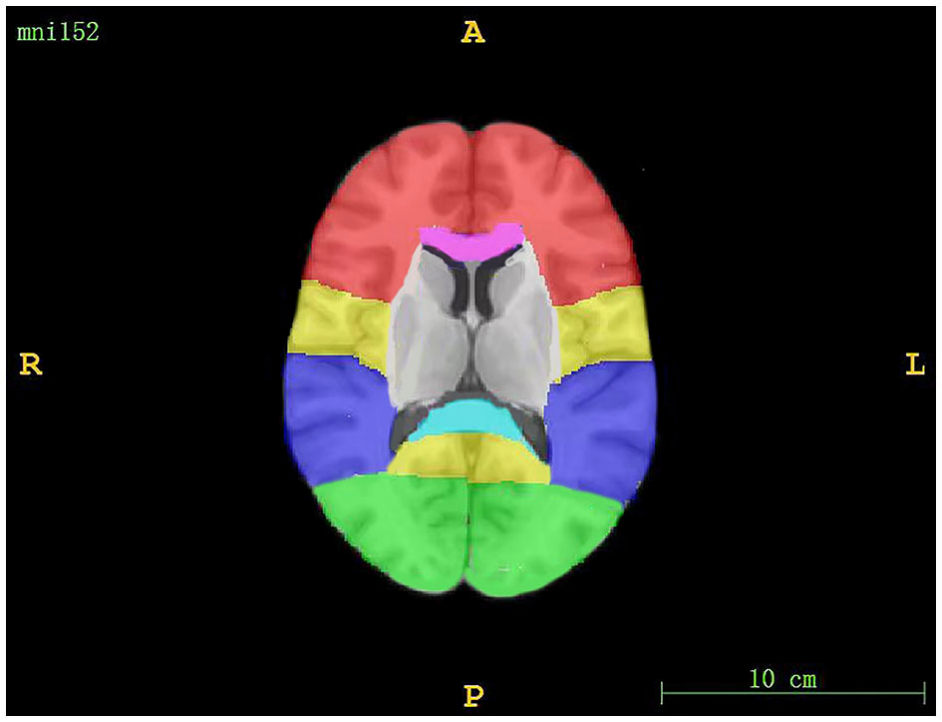
The segmentation of WMH volumes. The red region corresponds to the frontal lobe, the yellow region corresponds to the parietal lobe, the purple region corresponds to the temporal lobe, the green region corresponds to the occipital lobe, the pink region corresponds to the genu of the corpus callosum, and the blue region corresponds to the splenium of the corpus callosum.

The regions assessed for WMHs included periventricular white matter hyperintensity (PWMH) and deep white matter hyperintensity (DWMH). If the largest diameter of the WMH was adjacent to the ventricle, we defined it as PWMH; otherwise, it was considered to be DWMH (30). DWMH included WMH in the frontal, parietal, occipital, and temporal regions, as well as infratentorial regions (the brainstem and cerebellum). The segmentation of WMH volumes is shown in Figure 1. An existing lobar atlas was used to obtain regional WMH volumes for the above anatomic sites. The use of this atlas has been described previously (31, 32). PWMH included WMH in three regions, including frontal and occipital caps and bands (33).

The distribution of WMH volume was segmented using MATLAB R2022A (MathWorks, Natick, MA) via the lesion prediction algorithm (LPA; Schmidt, Chapter 6.1), an instrument implemented in the Lesion Segmentation Toolbox version 3.0.0 (www.statistical-modeling.de/lst.html) for SPM12. Large-scale regression models were used to calculate the lesion probability score for each voxel (34).

### Statistical analysis

Continuous variables are presented in the form of means ± standard deviation (SD) and were compared using the Student's t-test in the case of normally distributed variables. For non-normally distributed variables, regional WMH volumes are reported in the form of the median (maximum to minimum), other variables are reported in the form of the median (interquartile range), and one-way ANOVA was used for comparisons. Categorical data were examined using the chi-squared test or Fisher's exact test when appropriate and are reported in the form of the constituent ratio. To identify independent predictors of persistent cognitive impairment following lacunar stroke and build a prediction scale, a multivariate logistic regression model was constructed; variables with a *p* < 0.05 in the univariate analysis were included. SPSS 26.0 was used for statistical analysis.

## Results

### Baseline characteristics of patients

**Figure 2:**
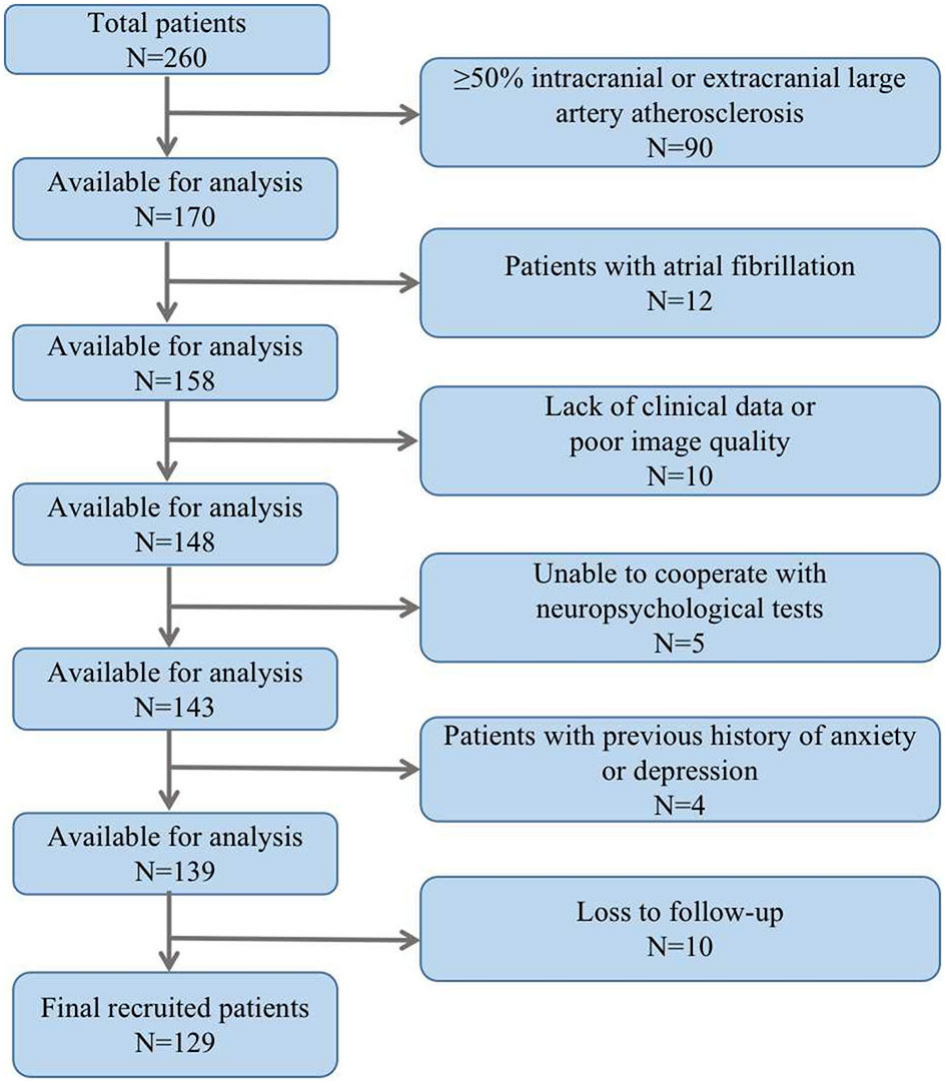
Patient selection.

**Figure 3:**
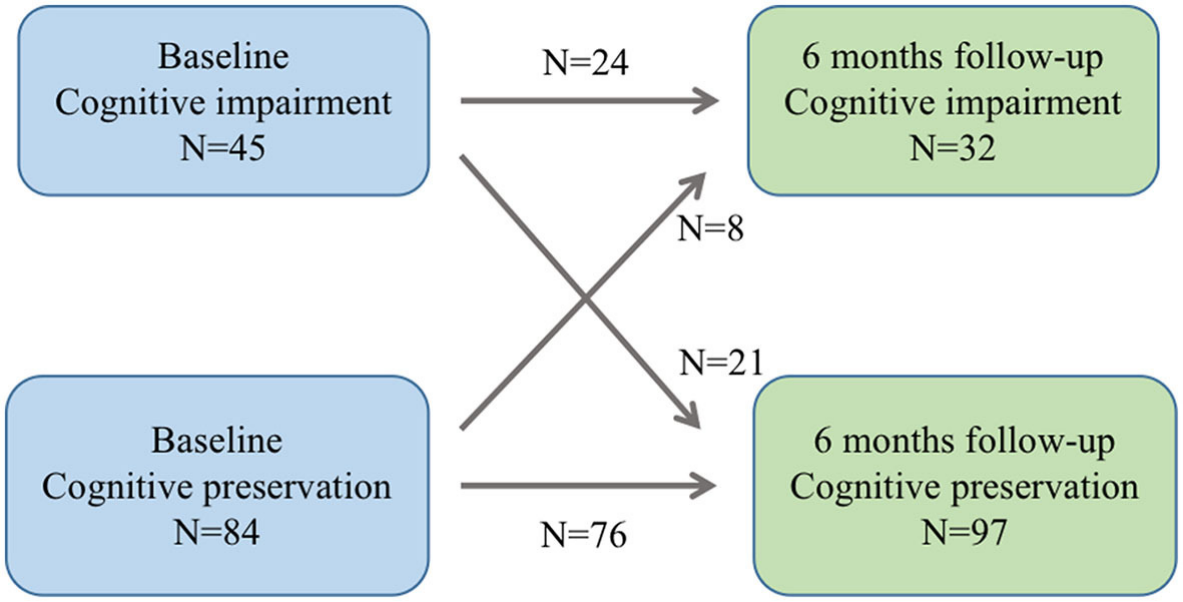
Cognitive trajectories regarding impairment (cognitive impairment and preservation from baseline to 6-month follow-up).

The study included a total of 129 ALS patients after evaluation (Figure 2), with 24.8% of patients experiencing cognitive impairment during the 6-month follow-up visit. Cognitive trajectories in terms of impairment (cognitive impairment) and preservation from baseline to 6-month follow-up are shown in Figure 3. The participants' characteristics and the differences between the groups with and without cognitive impairment are summarized in Table 1. Compared with the non-cognitive impairment group, the patients in the cognitive impairment group were older (69 ± 10.62 vs. 65 ± 9.33, *P* = 0.069) and had a higher incidence of depression (21.9% vs. 3.1%, *P* = 0.002). Patients with higher NIHSS scores (3 vs. 1, *P* = 0.004) and lower levels of albumin (37.31 ± 3.83 vs. 39.23 ± 3.33, *P* = 0.012) were more likely to experience cognitive impairment.

**Table 1:** Baseline characteristics of acute lacunar stroke patients with and without cognitive impairment.

**Subjects**	**Total patients (*n* = 129)**	**With cognitive impairment (*n* = 32)**	**Without cognitive impairment (*n* = 97)**	**Test statistic**	***P* value**
Age	67 ± 9.69	69 ± 10.62	65 ± 9.33	1.835	0.069^*^
Gender, male	80 (62.0%)	19 (59.4%)	61 (62.9%)	0.126	0.723
**Previous history**					
Cerebral infarction, *n* (%)	26 (20.2%)	8 (25.0%)	18 (18.6%)	0.621	0.431
Hypertension, n (%)	98 (76.0%)	25 (78.1%)	73 (75.3%)	0.108	0.742
Diabetes mellitus, n (%)	37 (28.7%)	11 (34.4%)	26 (26.8%)	0.674	0.412
NHISS score	2 (1–3)	3 (1–5)	1 (1–3)	−2.858	0.004^**^
Depression, *n* (%)	10 (7.8%)	7 (21.9%)	3 (3.1%)	9.389	0.002^**^
**Laboratory exam**					
White blood cells (10^9^/L)	6.15 (4.94–7.38)	6.15 (4.89–7.28)	6.15 (4.95–7.43)	−0.297	0.766
Neutrophils (10^9^/L)	3.80 (3.02–4.83)	4.02 (2.80–4.70)	3.76 (3.08–5.12)	−0.426	0.670
Lymphocytes (10^9^/L)	1.56 (1.18–1.98)	1.56 (1.15–2.07)	1.55 (1.18–1.92)	−0.460	0.646
Hemoglobin (g/L)	135 (124–144)	134 (122–144)	137 (124–145)	−0.697	0.486
Platelets (10^9^/L)	197.33 ± 55.06	195.90 ± 49.80	199.12 ± 56.73	−0.124	0.901
Total cholesterol (mmol/L)	4.36(3.75–5.05)	4.38 (3.69–4.94)	4.32 (3.75–5.05)	−0.402	0.688
Triglyceride (mmol/L)	1.38 (1.05–2.00)	1.31 (0.99–2.10)	1.38 (1.08–1.81)	−0.506	0.613
LDL-C (mmol/L)	2.60 ± 0.74	2.60 ± 0.63	2.58 ± 0.79	0.215	0.830
HDL-C (mmol/L)	0.95 (0.82–1.18)	0.91 (0.78–1.19)	0.96 (0.83–1.17)	−0.531	0.595
Blood glucose (mmol/L)	5.32(4.78–6.63)	4.97 (4.62–6.21)	5.35 (4.91–6.63)	−1.483	0.138
Total bilirubin (μmol/L)	14.80 (11.20–19.80)	16.20 (9.60–22.10)	14.70 (11.50–19.50)	−0.050	0.960
Uric acid (mmol/L)	324.55 ± 86.34	343.18 ± 85.59	317.63 ± 87.37	1.393	0.166
Urea (μmol/L)	5.30 (4.40–6.50)	5.60 (4.30–6.90)	5.20 (4.50–6.40)	−0.200	0.841
Prealbumin (mg/L)	248.98 ± 50.08	243.26 ± 49.34	251.20 ± 50.85	−0.499	0.618
Albumin (g/L)	38.75 ± 3.51	37.31 ± 3.83	39.23 ± 3.33	−2.561	0.012^**^
Homocysteine (μmol/L)	10.50 (8.93–12.18)	10.70 (9.20–12.00)	10.15 (8.70–12.61)	−1.147	0.251
C-reactive protein (mg/L)	1.96 (0.74–4.95)	2.88 (1.09–5.98)	1.78 (0.65–4.57)	−1.124	0.261
Plasma fibrinogen (g/L)	2.70 (2.21–3.15)	2.90 (2.42–3.14)	2.68 (2.21–3.19)	−0.808	0.419

SBP, systolic blood pressure; DBP, diastolic blood pressure; LDL-C, low-density lipoprotein; HDL-C, high-density lipoprotein; NIHSS, National Institutes of Health Stroke Scale; BI, Barthel Index; LAA, large artery atherosclerosis; CE, cardioembolism; SAO, small artery occlusion; SOE, stroke of other demonstrated etiology; SUE, stroke of other undetermined etiology. ^*^p < 0.1, ^**^p < 0.05.

In a qualitative analysis of CSVD imaging features among the two groups, EPVS (N>10) was found in 21 patients (65.6%) in the cognitive impairment group and 51 patients (52.6%) in the non-cognitive impairment group. Patients with higher total CSVD burden scores suffered cognitive impairment more often at the 6-month follow-up visit. Fazekas scores for periventricular WMH and deep WMH were 3 (2–3) and 2 (1–2) in the cognitive impairment group, respectively. In comparison, these scores were 1 (1–2) and 1 (0-1) in the non-cognitive impairment group. Patients with WMH in the genu of the corpus callosum (GCC) had a higher chance of exhibiting cognitive impairment (53.1% vs. 20.6%, *P* < 0.001) (Table 2).

**Table 2:** Qualitative analysis of imaging features in acute lacunar stroke patients with and without cognitive impairment.

	**Total patients (*n* = 129)**	**With cognitive impairment (*n* = 32)**	**Without cognitive impairment (*n* = 97)**	**Test statistic**	***P* value**
**Infarction lesions**					
Thalamus	14 (10.9%)	4 (12.5%)	10 (10.3%)	0.000	0.986
Basal ganglia/internal capsule	53 (41.1%)	14 (43.8%)	39 (40.2%)	0.125	0.724
Centrum ovale/corona radiata	56 (43.4%)	12 (37.5%)	44 (45.4%)	0.605	0.437
Medulla/midbrain/pons/ cerebellum	27 (20.9%)	8 (25.0%)	19 (19.6%)	0.426	0.514
**CSVD subtypes**					
Lacuna, n (%)	80 (62.0%)	21 (65.6%)	59 (60.8%)	0.235	0.628
Microbleeds, n (%)	23 (17.8%)	5 (15.6%)	18 (18.6%)	0.141	0.707
EPVS (N>10), n (%)	72 (55.8%)	21 (65.6%)	51 (52.6%)	5.215	0.074^*^
Periventricular WMH (Fazekas score, 0–3)	2 (1–3)	3 (2–3)	1 (1–2)	−3.805	< 0.001^**^
Deep WMH (Fazekas score, 0–3)	1 (0–1)	2 (1–2)	1 (0–1)	−2.454	0.014^**^
Genu of corpus callosum WMH, n (%)	37 (28.7%)	17 (53.1%)	20 (20.6%)	12.43	< 0.001^**^
Splenium of corpus callosum WMH, n (%)	31 (24.0%)	10 (31.3%)	21 (21.6%)	1.215	0.270
Total CSVD score (0–4)	2 (1–3)	2 (2–3)	2 (1–3)	−2.703	0.007^**^

WMH, white matter hyperintensity; CMB, cerebral microbleed; EPVS, enlarged perivascular spaces; sLI, silent lacunar infarction; CSVD, cerebral small vessel disease. ^*^p < 0.1, ^**^p < 0.05.

### Comparison of regional WMH volumes between the cognitive impairment and non-cognitive impairment groups

Total and regional WMH volumes were quantified for each group (Table 3). The acute lacunar infarction patients with cognitive impairment showed higher total WMH volume relative to the non-cognitive impairment group (median volume of WMH in the cognitive impairment group = 14.45 mm^3^; median volume of WMH in the non-cognitive impairment group = 5.52 mm^3^; *P* = 0.001). Statistically significant differences were also observed in the case of frontal (median volume was 2.96 mm^3^ in the cognitive impairment group vs. 0.52 mm^3^ in the non-impairment group; *P* = 0.006), parietal (median volume was 0.41 mm^3^ in the cognitive impairment group vs. 0.00 mm^3^ in the non-cognitive impairment group; *P* = 0.005), and periventricular WMH volume (median volume was 10.56 mm^3^ in the cognitive impairment group vs. 3.74 mm^3^ in the non-cognitive impairment group; *P* = 0.001). No differences were observed in the case of temporal, occipital, or subtentorial WMH volume (*P* > 0.05).

**Table 3:** Regional WMH volume in acute lacunar stroke patients with and without cognitive impairment.

**Regional WMH volume (cm^3^)**	**Total patients (*n* = 129)**	**With cognitive impairment (*n* = 32)**	**Without cognitive impairment (*n* = 97)**	**Test statistic**	***P* value**
Total	6.07 (0.00–64.08)	14.45 (0.00–64.08)	5.52 (0.32–38.58)	−3.419	0.001^*^
Frontal	0.62 (0.00–15.03)	2.96 (0.00–15.03)	0.52 (0.00–7.79)	−2.773	0.006^*^
Parietal	0.00 (0.00–6.99)	0.41 (0.00–6.99)	0.00 (0.00–6.89)	−2.835	0.005^*^
Temporal	0.00 (0.00–2.66)	0.00 (0.00–1.95)	0.00 (0.00–2.66)	−0.548	0.583
Occipital	0.00 (0.00–4.56)	0.00 (0.00–0.85)	0.00 (0.00–4.56)	−1.459	0.144
Periventricular	4.45 (0.00–40.70)	10.56 (0.00–40.70)	3.74 (0.00–30.46)	−3.371	0.001^*^
Subtentorial (brain stem/cerebellum)	0.00 (0.00–1.21)	0.00 (0.00–1.21)	0.00 (0.00–1.05)	−1.435	0.151

WMH, white matter hyperintensity, ^*^p < 0.05.

### Independent risk factors for cognitive impairment and the prediction scale

Multivariable logistic regression analysis showed that WMH at the GCC, depression, NIHSS score, and albumin at admission were important determinants of long-term cognitive impairment after ALS (Table 4). Regarding regional WMH volume, only frontal WMH volume was a significant predictor of cognitive impairment, even though both parietal and periventricular WMH volume were also entered into the regression model. Thus, depression (OR = 6.252, *P* = 0.029), NIHSS score (OR = 1.24, *P* = 0.011), albumin level (OR = 0.841, *P* = 0.032), WMH at the GCC (OR = 3.1, *P* = 0.033), and frontal WMH volume (OR = 1.18, *P* = 0.04) were used to construct a cognitive prediction scale.

**Table 4:** Multivariable logistic regression for cognitive impairment in lacunar stroke patients.

	**B**	**OR (95% CI)**	***P* value**
Age (years)	0.028	1.029 (0.973–1.087)	0.317
Depression	1.833	6.252 (1.213–32.232)	0.029^*^
NIHSS score	0.215	1.240 (1.050–1.464)	0.011^*^
EPVS (N > 10)	−0.042	1.062 (0.391–2.882)	0.906
Albumin (g/L)	−0.173	0.841 (0.718–0.985)	0.032^*^
Frontal WMH volume (cm^3^)	0.166	1.180 (1.008–1.382)	0.040^*^
Parietal WMH volume (cm^3^)	0.06	1.062 (0.769–1.466)	0.715
Periventricular WMH volume (cm^3^)	−0.001	0.998 (0.902–1.104)	0.970
WMH at genu of corpus callosum	1.132	3.100 (1.095–8.775)	0.033^*^

NIHSS, National Institutes of Health Stroke Scale; BI, Barthel Index; CSVD, cerebral small vessel disease. ^*^p < 0.05.

## Discussion

This was a prospective study on PSCI, with a 6-month follow-up period, in acute lacunar stroke patients. In this study, we identified depression, plasma albumin, and NIHSS score at admission as independent risk factors for the occurrence of persistent PSCI in the 6-month time course. We further found a significant positive correlation between baseline frontal WMH volume and long-term cognitive impairment, whereas periventricular WMH volume did not correlate with cognition, although this volume was higher among the cognitive impairment group than the non-cognitive impairment group. The study also demonstrated that the occurrence rate of GCC WMH at baseline was statistically significantly different between the cognitive impairment and non-cognitive impairment groups.

Small vessel-related ischemic stroke, or acute lacunar stroke, is one of the common stroke subtypes (35, 36). Although these patients have a small infarct, they usually carry a higher risk of cognitive impairment than would be expected (36). Another recent review summarized domain-specific cognitive impairment after lacunar stroke; the results showed that such dysfunction was comprehensive rather than domain-specific (5). Furthermore, cognitive impairment after lacunar stroke is associated with recurrent stroke and death. Acute lacunar stroke is not solely lacunar infarct; it is a part of the spectrum of CSVD, which is a systemic pathology that influences the brain extensively. The presence of cognitive impairment after lacunar stroke is a symbol of the unmasked microangiopathy (37, 38).

WMH is widely recognized as an independent risk factor for cognitive impairment. A meta-analysis showed that WMH is associated with substantially increased risks of long-term cognitive impairment and all-cause dementia (7). WMH may cause cognitive impairment via several mechanisms. First, white matter lesions may disrupt the subcortical neural networks, such as the frontal subcortical circuit, the frontal thalamus circuit, and the dorsolateral prefrontal and cingulate circuits (39–41). Second, WMH has been shown to be related to dementia-related pathological processes, including Wallerian degeneration or lack of myelin occurring secondary to neuronal loss (42). Furthermore, WMH may contribute to the accumulation of amyloid-β proteins, which is secondary to the impaired drainage of extravascular proteins along blood vessels (43).

Regarding the influence of CSVD-related WMH on cognitive impairment in ALS patients, our study indicated that baseline Fazekas grades of both periventricular WMH and deep WMH were relevant; this result was similar to those of other studies (44). Measurement of the severity of WMH is mostly based on semiquantitative visual rating scales such as the Fazekas visual grade or Scheltens grade (45). Many population-based studies have identified an association between WMH severity and cognitive decline. Recently, volumetric measures of WMH were employed to assess contributions to cognitive impairment sensitively and reliably, which added additional value to WMH grades. Cross-sectional studies that have used WMH volume have suggested that a higher volume of WMH is associated with greater impairments in global cognition as well as perceptional speed, memory, processing speed, and executive function; however, these studies have showed a weak evidential effect (46–48). While some studies have investigated the effects of total WMH volume on cognition, the regional specificity of WMH volume has rarely been explored. Sheline et al. reported that the strategic location of WMH is critical in late-life depression (49). Another study discovered that periventricular hyperintensity in the frontal caps and occipital WMHs were strong predictors of balance and gait impairment (50).

This study focused on the effect of baseline regional WMH volume on long-term cognitive impairment in ALS patients. We found that frontal WMH volume was statistically significantly associated with long-term cognitive impairment in ALS patients after adjustment for NIHSS score, albumin, and depression. Frontal lobe WMH volume is correlated with age, but this is not true for other lobes, such as the temporal lobes (51). Furthermore, CSVD usually causes underlying dysfunction of frontal-subcortical circuits; a higher frontal WMH volume may represent a preexisting strike against cognitive reserves. Frontal lobe WMHs have been reported to be associated with cognition in certain other populations, such as PD patients (52), patients with objectively defined subtle cognitive decline (Obj-SCD) (53), and healthy older adults (22). However, the findings of the current study were contrary to those of a previous study suggesting that WMH volume in the parietal lobe, rather than the frontal lobe, is a predictor of the timing of onset of dementia in the community (21). Therefore, more systematic and large-scale studies are needed in the future. Regional WMH volume would provide more quantitative information for clinical research, and the researchers could obtain WMH topography using regional WMH volume in order to better understand the underlying mechanisms.

In this study, we additionally found that the baseline presence of GCC WMH was independently related to persistent cognitive impairment in ALS patients. Pozorski et al. found that impaired memory in PD patients was associated with a higher probability of WMH in and adjacent to the GCC (52). However, Garnier-Crussard et al. showed that WMH in the larger splenium of the corpus callosum (SCC) is a core feature in Aβ-positive AD patients (54). This differs from the findings presented here. Owing to the extensive structural connections of the corpus callosum with the frontal, temporal, parietal, and occipital lobes of the bilateral cerebral hemispheres, it plays a crucial role in the functions of movement, sensation, language, visual discrimination and perception, auditory localization, and accommodation and coordination of information. A study by Raghavan et al. has reported reduced fractional anisotropy (FA) of the GCC to be a cerebrovascular disease marker and a predictor of longitudinal cognitive impairment with the use of diffusion tensor imaging (DTI) (55). Additionally, several lines of evidence indicate the existence of a relationship between CC and vascular disease (56), suggesting that the GCC is the region most vulnerable to the effects of vascular disease, as the SCC would be an important hub in AD, located in the vicinity of brain areas that are the most sensitive to AD pathology (57).

Our study has both strengths and limitations. A main strength was the homogenous sample of recruited patients with symptomatic CSVD-related lacunar stroke. There have been a few comparable studies analyzing the effect of baseline CSVD on PSCI in cohorts who have experienced heterogeneous forms of stroke with different etiologies and infarct lesion sizes (58–61). Moreover, our longitudinal design allowed assessment for long-term cognitive impairment.

However, our study also had several limitations. First, this was a monocentric and small-sample study. We defined several exclusion criteria, which might affect our results and conclusions. Therefore, sampling bias and possible selection bias should be considered. Second, some participants were lost to follow-up, and no comparison of the baseline characteristics was made between the patients lost to follow-up and those included in the study. Thus, withdrawal bias could not be ruled out. Third, cognitive evaluation was performed only using the MoCA scale for global cognitive status; more extensive neuropsychological assessments for distinct cognitive domains should be carried out. Fourth, we did not know the patients' pre-stroke cognitive status, although we excluded those who had severe cognitive impairment or dementia before falling ill.

Furthermore, several patients were excluded for reasons of aphasia or disturbance of consciousness at admission, and yet, these patients may suffer from more severe stroke. Therefore, patients included in our study may have had relatively mild stroke attacks. Finally, the cross-sectional and observational design of the study prevented us from assessing the dynamic evolution of WMH.

## Conclusion

Our study is one of a few existing studies to observe an association between volumetric analyses of regional WMH and cognitive impairment, providing new evidence that frontal WMH volume and the presence of WMH at the GCC at baseline are the core features of long-term cognitive impairment after ALS. Carefully established insights into regional WMH via brain magnetic resonance may greatly help to diagnose ALS patients with a higher risk of long-term cognitive impairment.

## Data availability statement

The raw data supporting the conclusions of this article will be made available by the authors, without undue reservation.

## Ethics statement

The studies involving humans were approved by Research Ethics Committee of First Affiliated Hospital of Soochow University. The studies were conducted in accordance with the local legislation and institutional requirements. The participants provided their written informed consent to participate in this study.

## Author contributions

TL: Data curation, Investigation, Writing—original draft. MY: Investigation, Writing—original draft. GY: Methodology, Software, Writing—review and editing. SD: Methodology, Writing—review and editing. YZ: Data curation, Methodology, Writing—review and editing. YQ: Methodology, Writing—review and editing, Investigation. DD: Formal analysis, Investigation, Writing—review and editing. MZ: Software, Supervision, Writing—review and editing. QF: Supervision, Validation, Visualization, Writing—review and editing.
